# Molecular and Biochemical Characterization, Antimicrobial Activity, Stress Tolerance, and Plant Growth-Promoting Effect of Endophytic Bacteria Isolated from Wheat Varieties

**DOI:** 10.3390/microorganisms10010021

**Published:** 2021-12-23

**Authors:** Dawood Shah, Mohammad Sayyar Khan, Shahkaar Aziz, Haidar Ali, Lorenzo Pecoraro

**Affiliations:** 1 Institute of Biotechnology and Genetic Engineering, The University of Agriculture Peshawar, Peshawar 25000, Pakistan; dawoodshah616@gmail.com (D.S.); sayyarkhankazi@aup.edu.pk (M.S.K.); shahkaaraziz@aup.edu.pk (S.A.); haider@aup.edu.pk (H.A.); 2 School of Pharmaceutical Science and Technology, Tianjin University, 92 Weijin Road, Tianjin 300072, China

**Keywords:** bacterial endophytes, wheat varieties, phylogeny, biochemical analysis, antimicrobial activity, stress tolerance, plant growth-promoting ability

## Abstract

Endophytic bacteria have been utilized as an alternative source to chemical fertilizers and pesticides to enhance plant productivity and defense mechanisms against biotic and abiotic stress. Five endophytic bacterial strains were isolated from the seeds of three different Pakistani wheat varieties (Ghaneemat-e-IBGE, Atta-Habib, and Siren). The isolated strains AH-1, S-5, S-7, GI-1, and GI-6 showed phylogenetic similarity with *Bacillus altitudinis*, *B. aryabhattai*, *B. wiedmannii*, *Pseudomonas aeruginosa*, and *Burkholderia gladioli*, respectively. All strains showed catalase activity (except AH-1) and Indole-3-acetic acid production, with the highest concentration (16.77 μg·mL^−1^) found for GI-6, followed by S-5 (11.5 μg·mL^−1^), nitrogen assimilation (except S-7), phosphorus solubilization (except S-7 and AH-1), and ability to produce siderophores, with maximum productions for GI-6 (31 ± 3.5 psu) and GI-1 (30 ± 2.9 psu). All five analyzed strains possessed antimicrobial activity, which was particularly strong in GI-6 and S-5 against *Klebsiella pneumonia*, *Escherichia coli*, and *Bacillus subtilis*. Increasing salinity stress with NaCl negatively affected the bacterial growth of all isolates. However, strains GI-6 and S-5 showed salt tolerance after three days of incubation. A drought tolerance test resulted in a negative impact of poly ethylene glycol on bacterial growth, which was, however, less pronounced in GI-6 strain. The GI-6 strain revealed growth-promoting effects on inoculated wheat plants.

## 1. Introduction

In the wake of the growing world populations and confronting challenges of climate change, eco-friendly applications of biotechnology and plant breeding to enhance agriculture productivity are crucial. Usage of manures, pesticides, and chemical fertilizers are traditional methods to increase soil fertility and crop yields that also negatively impact the environment through fertilizers adsorption, runoff, and hazardous accumulation of chemicals, such as cadmium, in soil [1]. Plant growth-promoting (PGP) microbes are an effective and environmentally sustainable alternative for replacing chemical fertilizers. Over the last few years, researchers have increasingly focused on microbial bio-inoculants as bio-fertilizers and bio-pesticides for sustainable agriculture [2].

Endophytes are microbes that colonize plant tissues without causing any disease symptoms. In typical conditions, endophytes play a vital role in the development and growth of host plants by enhancing the assimilation of nutrients and the production of secondary metabolites, which protect the plant from various disease-causing pathogens. Endophytic microbes such as bacteria and fungi, form a network around their host plants and protect them from unfavorable climatic and other environmental changes [3]. Endophytes enhance plant growth through the fixation of atmospheric nitrogen, uptake of phosphorus, potassium, and zinc, the development of Fe^3+^-chelating molecules and the secretion of different phytohormone (phytostimulation), such as auxins, ethylene, gibberellins, and cytokines [4]. In the endophytic interaction, microbes have an indirect function to control the harmful effect of phytopathogens on plant and soil health via the synthesis of innumerable substances, including immunosuppressants, antibiotics, bio-control agents, hydrogen cyanide (HCN), and ammonia [5].

Several bacteria, including *Bacillus* sp., *Pseudomonas* sp., *Acinetobacter* sp., *Azotobacter* sp., and *Azospirillum* sp., generate phytohormone-like cytokinins, indoleacetic acid, octadecanoid acids, and gibberellin compounds that imitate the action of jasmonates and contribute to the survival of the plants. Endophytic bacteria synthesize organic acids to solubilize phosphorus, thus increasing phosphorus supply to the plant [6]. 

Wheat (*Triticum* spp.) is one of the world’s most valuable crops, providing more calories and proteins than any other grown crop. It is a staple food for over 35% of the world’s population [7]. It is cultivated by approximately 80% of Pakistani farmers, accounting for nearly 40% of the total cultivated areas in Pakistan. It is planted on 9151 thousand hectares of land in the country, yielding 25,980 thousand tons of wheat grains at an average yield of 4054 kg ha^−1^ [8].

Drought is a global issue that affects almost every wheat-producing area and causes extreme osmotic stress. Global warming is the key factor that persistently increases the problem of drought and associated risk to crop yields [9]. Soil salinization, which threatens around 20% of arable cropland worldwide, is another source of high osmotic stress for cultivated plants [10]. Soil salinization is becoming a dominant problem in agriculture, with half of all arable cropland expected to be affected by 2050 [10,11]. During the later tillering phases, drought and osmotic stress were found to reduce yield by lowering kernel quantity and slowing plant recovery [12]. Drought negatively affects many additional plant metrics, such as leaf area index, dry matter accumulation, and net absorption rates [13]. Additionally, drought can disrupt metabolic processes such as photosynthesis and impair sugar synthesis, which is essential to drive the wheat yield [14]. A decrease in kernel number and weight was found when salt stress was applied after terminal spikelet growth [15]. Because of the low water content, high salinity disrupts plant selective ion absorption at cellular level, affecting nutrient availability [16].

The molecular identification and ecological characterization of plant-associated microbes are critical to understand the exact role these microorganisms play, their interactions with plants, and to develop effective biotechnological applications using endophytic strains to improve agricultural production [17]. This study aimed to assess the diversity of endophytic bacteria in different wheat varieties collected in Pakistan and to characterize the plant growth-promoting, drought, salt, and disease-resistance traits of the isolated bacterial strains. The analyzed endophytic microbes could be used as biofertilizers and biopesticides to support the development of sustainable agriculture.

## 2. Materials and Methods

### 2.1. Isolation of Endophytes 

The seeds of three different indigenous wheat (*Triticum aestivum*) varieties from Pakistan, namely, Ghaneemat-e-IBGE, Atta-Habib, and Siren were used to isolate endophytic bacteria. Seeds were surface sterilized using 70% ethanol for 30 s, then washed with 15% sodium hypochlorite solution for 2 min, followed by rinsing with 70% ethanol for 30 s. Finally, the seeds were thoroughly rinsed three times with double distilled water. Complete disinfection was checked after incubating 1 mL of the last seed rinsing water in 10 mL of liquid broth (LB) media for 48 h. Ten intact disinfected seeds were placed on Petri dishes containing LB and incubated at 24 °C. After 7 days of incubation, the appeared colonies were selected and characterized morphologically. The selected colonies were sub-cultured following the method by [18] to obtain pure cultures. The morphologically distinct endophyte pure colonies were stored at 4 °C.

### 2.2. Identification of Endophytes

The isolated endophytes were characterized by colony morphology, shape, color, texture, and growth according to the method described by [19]. For Molecular identification, the isolated microbes were cultured in LB broth at 30 °C for 24 h in a shaker at 220 rpm, and then centrifuged at 4000 rpm. The cell pellets were used for bacterial genomic DNA isolation according to optimized protocols [20]. The isolated endophytes were identified based on the amplification of the 16SrRNA gene. A 1500-bp sequence was amplified from genomic DNA using the primers P027F and 1378R specific for the 16S ribosomal RNA genes. In a 25 μL PCR mixture, 1 μL (0.5–10.0 ng) template DNA, 0.2 μM each of primers P027F (5′-GAGAGTTTGATCCTGGCTAG-3) and 1378R (5′-CGGTGTGT ACSSGGCCCG GGAACG-3′), 200 μM each dNTP, 10 × buffer, 2 mM MgSO_4_, and 1 U high-fidelity KOD Taq DNA polymerase were used [21]. The amplification program was as follows: initial denaturation for 4 min at 94 °C; 30 cycles of denaturation for 30 s at 94 °C, annealing for 1 min at 63 °C, extension for 1 min at 68 °C, and a final total extension for 7 min at 68 °C. The PCR was performed in a thermocycler programmed as follows: 94 °C for 2 min; 35 cycles of 94 °C for 45 s, 55 °C for 60 s, 72 °C for 60 s and final extension for 72 °C for 10 min. The PCR products were purified and sent to Macrogen, Korea Co. Ltd. for sequencing. BLAST searches of the 16S rRNA sequences obtained from the isolated bacterial strains were performed in the NCBI database. The homologous 16S rRNA sequences were aligned using the multiple-sequence alignment tool “CLUSTAL-W” in MEGA 7 software [22]. The phylogenetic tree was constructed using the neighbor-joining and maximum likelihood methods [23].

### 2.3. Identification of Siderophore

The isolated endophytes were characterized for siderophore production using a modified standard method for inoculating bacterial cells in liquid 284 medium with a chrome azurol sulphonate (CAS) shuttle solution, where the siderophore formation was stimulated. In microcentrifuge tubes, approximately 50 μL of bacterial suspension in MgSO_4_ (10 mM) was injected into 800 μL of 284 media with three different iron concentrations. Iron concentrations of 0 μM, 0.25 μM, and 3 μM Fe (III) citrate were used. The samples were shaken and incubated at 30 °C for 5 days (150 rpm). After incubation, 100 μL of the blue CAS reagent were added, and the tubes were kept at room temperature for 4 h. Following this step, the shift in color from blue to orange/yellow was regarded as a positive. Both samples had their siderophore concentrations estimated at 630 nm. The siderophore quantities were calculated using the formula: percent of siderophore units (psu) Ar-As/Ar 100, where “Ar” represents the absorbance of the reference (CAS reagent) and “As” represents the absorbance of the sample at 630 nm. In the bacterial isolates, a qualitative test using the CAS agar assay confirmed siderophore formation. In Minimal Media 9, the FeCl_3_ and HDTMA-containing CAS solution was combined with 20% glucose, CAS amino acid solution, and bacto-agar (MM9). Bacterial isolates were inoculated onto CAS agar plates and kept at 28 °C in the dark for two weeks. The appearance of yellow/orange halos around the colonies confirmed the siderophore production.

### 2.4. Indole-3-Acetic Acid Production 

The production of indole-3-acetic (IAA) was assayed according to the modified method of Gordon and Weber (1951). It was measured by growing isolates in LB media overnight at 28 °C with shaking at 220 rpm for 24 h. A total of 2 mL of supernatant was combined with two drops of orthophosphoric acid and an equal volume of Salkowski’s reagent after centrifugation of cell culture [24]. Furthermore, the optical density was measured using a UV spectrophotometer at 535 nm, and the concentration was calculated.

### 2.5. Phosphates Solubilization

The potential strains were streaked using a modified method from [9] for phosphate solubilization on Pikovskaya’s agar medium, which contains (per liter): 0.5 g yeast extract, 10 g dextrose, 5 g Ca_3_(PO_4_)_2_, 0.5 g (NH_4_)_2_SO_4_, 0.2 g KCl, 0.1 g MgSO_4_·H_2_O, 0.0001 g MnSO_4_·H_2_O, 0.0001 g FeSO_4_·H_2_O. Fifteen strains that produced a clear zone around the colonies after 3 days of incubation at 28 °C were considered positive. The phosphate solubilization index was calculated by measuring the colony diameter and the halo zone diameter, using the formula: Phosphate Solubilization Index (SI) = (Colony diameter + Halo zone diameter)/Colony diameter.

### 2.6. Catalase Test

The catalase presence in the isolates was checked by placing a drop of 3% H_2_O_2_ and adding the bacterial colony on a glass slide. Bubble formation was regarded as positive, while the absence of bubbles or a few scattered bubbles was regarded as negative [25].

### 2.7. Nitrogen Fixation

Nitrogen fixation capacity was determined by growing the isolated endophytic bacteria in nitrogen-free Ashby medium and Jensen medium [26]. The bacterial strains were inoculated in 5 mL of Ashby medium (without agar) in a 45 mL test tube and cultured for 7 days on a rotary shaker (125 rpm) at 28 °C. The bacterial colony growth was also tested on the Jensen medium at 28 °C for 4 days. 

### 2.8. Antimicrobial Activity 

The isolated endophytes were used for antimicrobial activity tests against the pathogenic bacteria *Klebsiella pneumonia*, *Escherichia coli*, and *Staphylococcus aureus*, and the non-pathogenic bacterium *Bacillus subtilis*. The disc diffusion method was applied to perform the antimicrobial bioassays, using two-day-old cultures of the selected pathogenic bacterial strains. The endophytic bacterial culture was spot-inoculated at three equidistant points of the LB agar plate, about 2.5 cm away from the middle, with 5, 10, and 15 μL doses. A 6 mm microbial plug was mounted in the plate’s middle, and the plates were incubated at 28 °C. Plates containing endophytes without bacterial inoculation were used as a negative control. The pathogen’s growth against the endophytic bacterial strain was monitored on a regular basis. The zone of inhibition of pathogenic growth was measured after the control plates reached the plate edges. The following formula was used to calculate the pathogen’s growth inhibition: ((C-T)/C) 100, where C represents the test pathogen’s radial growth in the control plates (mm) and T represents the test pathogen’s radial growth in the test plates. Ciprofloxacin was used a positive control.

### 2.9. Analysis of Endophytic Strain Tolerance to Drought and Salt Stresses

The growth of the selected bacterial isolates was analyzed at various stress levels following the method by Pirhadi et al. [27]. Trypticase soy broth (TSB) was supplemented with different poly ethylene glycol (PEG) concentrations, including the 0, 2.5%, 5%, 7.5%, and 10% PEG, to screen the isolates for drought-stress tolerance. The different media were inoculated with isolated strains’ overnight grown broth cultures with an adjusted optical density (OD) of 0.1 at 600 nm. The growth of the isolates at various stress levels was estimated by measuring the OD at 600 nm after incubation at 28 °C for 24 h [28]. The salt tolerance capacity of each isolate overnight culture was dropped in triplicate on nutrient agar plates containing different concentrations of NaCl (0, 50, 100, 150, and 200 mM) and incubated at 30 °C for three days. The colony’s diameter was measured regularly. Colony growth was compared, and a control inoculum was performed without any salt added. The percentage reduction in growth in salt-amended media was determined using the following formula:

(100 × A − B/A)(1)

where A represents colony diameter growth in the control plate, in mm, of the isolate and B represents colony diameter growth in the salt amended plate in mm [26].

### 2.10. Pot Experiment for Evaluating Plant Growth-Promoting Effect of Isolated Endophytic Bacteria

A completely randomized design was used to analyze the isolated endophyte strain GI-6 for growth-promoting effect on the *Triticum aestivum* cultivar Imdad in a controlled greenhouse environment. The endophyte was cultured overnight in 5 mL LB, transferred in 50 mL LB broth for 24 h at 30 °C with 220 rpm shaking, re-inoculated in 400 mL LB and incubated for another 24 h at 30 °C. A spectrophotometer was used to determine the culture’s optical density (OD) (600 nm). The culture was then diluted ten times with tap water, and seeds of the wheat variety (Imdad) were soaked in the diluted culture for around 40 min before being used as controls. Non-inoculated seeds of the studied wheat variety were soaked in ten times-diluted LB broth. Morphological observation of agro-phenotype characters of the cultivar, including plant height, spike length, leaf length, root length, and number of grains per spike, was performed.

### 2.11. Graphical and Statistical Analysis

The obtained data were statistically analyzed through ANOVA, MEGA 7, BLAST, SPSS, and Pub gene 3.2 software packages.

## 3. Results

**Figure 1:**
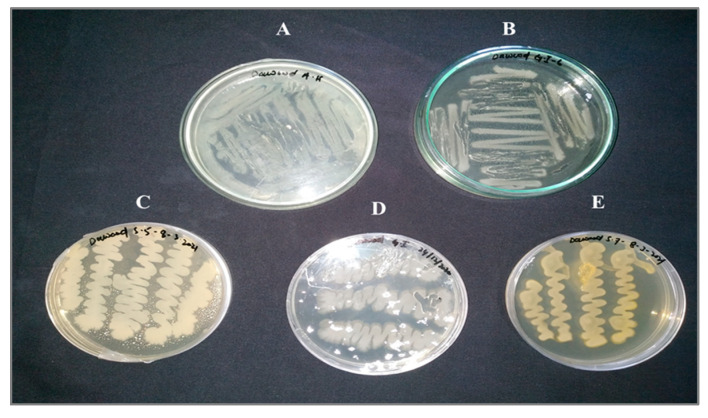
Colony morphology of the analyzed endophytic bacterial strains on LB agar plates: (**A**) Atta Habib (AH-1), (**B**,**C**) Ghaneemat-e-IBGE (GI-1, GI-6), (**D**,**E**) Sirin-5, Sirin-7.

**Figure 2:**
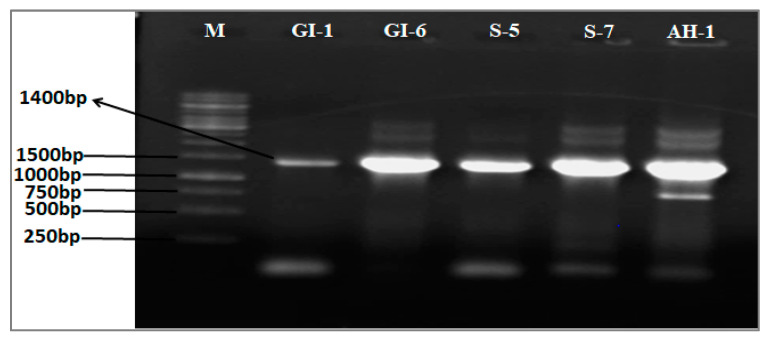
Agarose gel analyses (1% agarose, *w*/*v*) of amplified 16S RNA of the isolates used in the study. (Lane M = marker; lane 1 = GI-1; lane 2 = GI-6; lane 3 = S-5; lane 4 = S-7; lane 5 = AH-1).

In the current study, five endophytic bacteria were isolated from the seeds of the different investigated wheat varieties. The isolated strains, named AH-1, S-5, S-7, GI-1, and GI-6, were morphologically and molecularly identified as *Bacillus altitudinis*, *B. aryabhattai*, *B. wiedmannii*, *Pseudomonas aeruginosa*, and *Burkholderia gladioli*, respectively (Figure 1 and Figure 2). 

**Figure 3:**
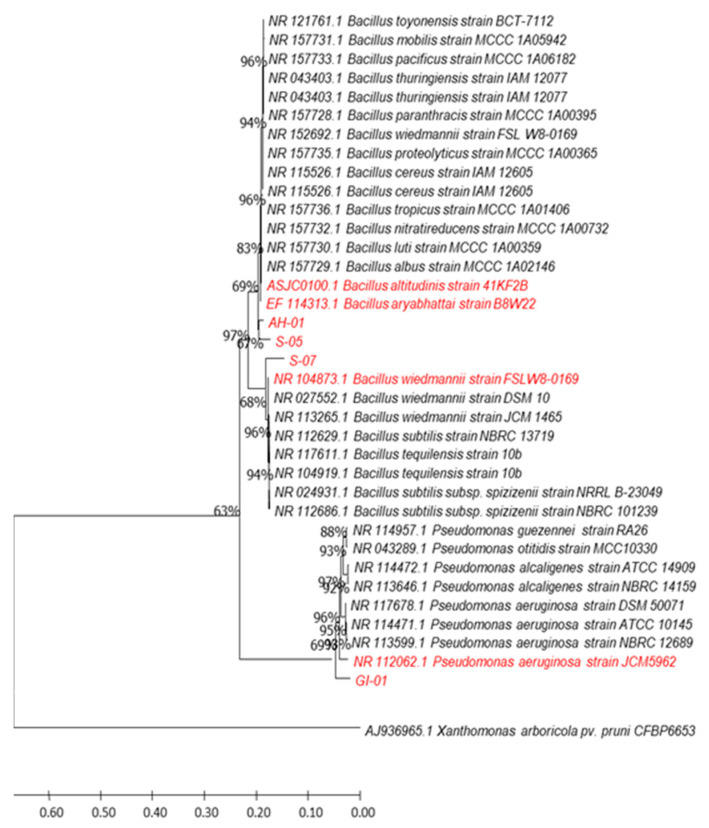
The 16S rRNA gene sequences of bacteria GI-01, S-07, S-05, and AH-01, as well as related bacteria in the genera *Pseudomonas* and *Bacillus*, were used to create a maximum-likelihood phylogenetic tree. The phylogenetic relationship was deduced using the Tamura-Nei model and the maximum likelihood approach (1993). Bootstrap values are expressed as a percentage of 1000 replicates; values less than 50% are not given. *Xanthomonas arboricola* pv. *pruni* strain CFBP6653, AJ936965.1 was used as outgroup. Sequences from the analyzed endophytic bacterial strains and from their best matches are in red.

Molecular analysis indicated that the isolated strains belonged to the genera *Bacillus*, *Pseudomonas*, and *Burkholderia*. Based on BLAST search in NCBI, the strains S-5 and S-7 showed 100% similarity with *Bacillus aryabhattai* and *B. wiedmannii*, respectively (Table 1). The strain AH-1 exhibited 99.97% similarity with *Bacillus altitudinis*, while GI-1 and GI-6 showed similarity with *Pseudomonas aeruginosa* (99.80%) and *Burkholderia gladioli* (99.70%), respectively (Table 1). The maximum-likelihood approach was used to determine the phylogenetic relationship between the selected database sequences. Isolate AH-1 was found in the same cluster with isolate S-5, and was closely related to *Bacillus altitudinis* and *B. aryabhattai*. Isolate S-7 was close to *B. wiedmannii*, while isolates GI-1 showed phylogenetic similarity with *Pseudomonas aeruginosa* (Figure 3). 

**Figure 4:**
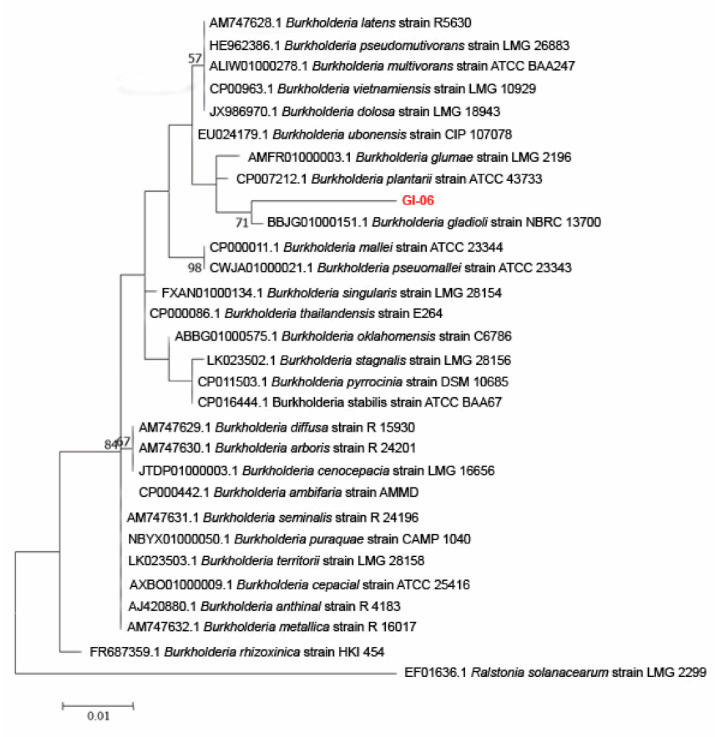
Maximum-likelihood phylogenetic tree constructed using the 16S rRNA gene sequences of the strain GI-06 and selected database sequences in the genus *Burkholderia*. The phylogenetic relationship was deduced using the Tamura-Nei model and the maximum likelihood approach (1993). Bootstrap values are expressed as a percentage of 1000 replicates; values less than 50% are not given. *Ralstonia solanacearum* strain LMG2299, EF016361.1 was employed as outgroup. A total of 0.01 substitutions per nucleotide location are shown in the bar. A total of 31 nucleotide sequences were examined. Gaps and missing data were removed from all positions. The total number of places in the final dataset was 95. The sequence from the analyzed endophytic bacterial strain is in red.

Similarly, a maximum-likelihood phylogenetic tree was constructed for *Burkholderia* genus, showing that the isolate GI-6 formed a clade with the *B. gladioli* with a bootstrap value of 71 (Figure 4).

### 3.1. Biochemical Characterization of Endophytic Bacterial Isolates

#### 3.1.1. Catalase Test

Four out of the five analyzed endophytic bacterial strains showed positive results of catalase activity. The positive catalase activity was shown by GI-6, S-5, GI-1, and S-7, while AH-1 was catalase-negative (Supplementary Figure S1 and Table S1).

#### 3.1.2. Indole Acetic Acid Production Potential of the Isolated Strains

**Figure 5:**
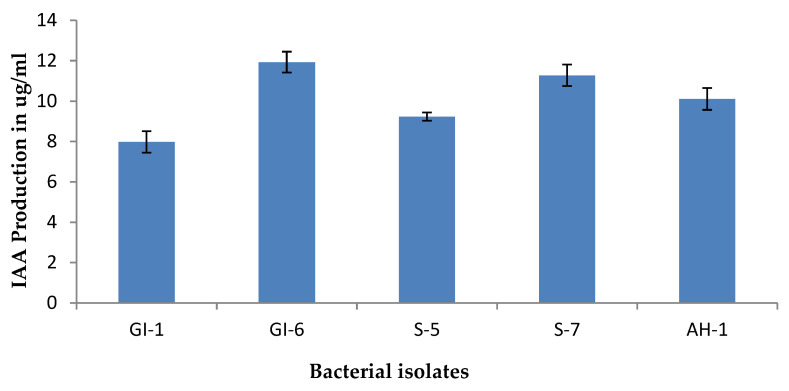
IAA production of the isolated strains. Data are averages ± SD values.

Isolate GI-6 showed the highest IAA activity (16.77), while the lowest was recorded for the strain GI-1 (8.45), as shown in Figure 5 and Supplementary Figure S2. 

#### 3.1.3. Siderophore Production Potential of the Isolated Endophytic Bacterial Strains

**Figure 6:**
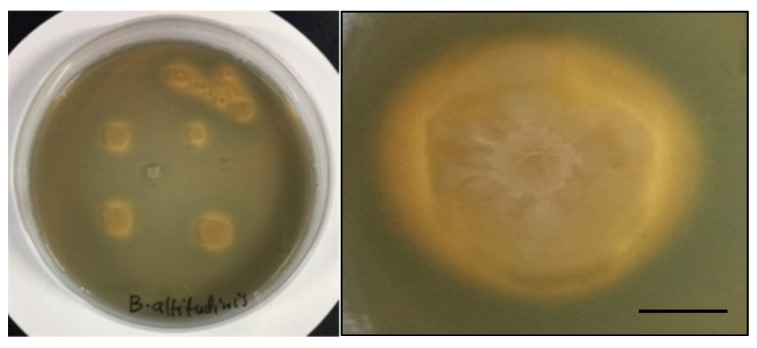
Siderophore production activity of the isolated strains. Overview of various colonies growing in a capsule (**left**) and detail of a single colony showing the typical yellow-orange halo (**right**) are provided. Scale bar 2 mm.

Two endophytic strains out of five grew on CAS agar medium. Isolates GI-6 and AH-1 showed promising results for siderophore production in the form of an orange halo around the colonies. Siderophore development was considered negative in isolates that showed no color change around the edge of the colonies (Figure 6). 

**Figure 7:**
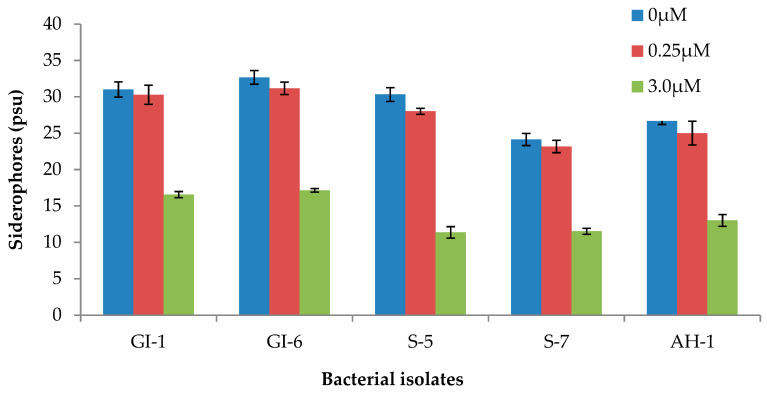
Siderophore production by the bacterial isolates at various Fe (III) citrate concentration. Data are averages ± SD.

The formation of a yellow-to-orange halo around the growth edge indicated that the siderophores produced by the bacterial strains started iron chelation. In fact, the capacity of the siderophore to remove the iron from the dye complex results in the observed color change. By sequestering usable iron, these siderophore-producing endophytes minimize the accessibility of iron to iron-requiring phytopathogens. As a result, they indirectly boost plant growth. The ability of the analyzed strains to produce siderophores at various iron citrate concentrations was checked. All strains produced siderophores, and the concentration varied among the strains. However, increasing the Fe (III) citrate concentration in the medium negatively impacted the siderophore accumulation, which was more pronounced in strains, S-5, S-7, and AH-1 (Figure 7 and Supplementary Table S1).

#### 3.1.4. Phosphorous Solubilization

**Figure 8:**
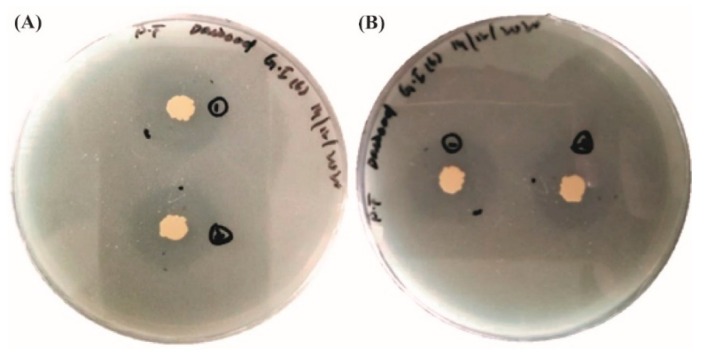
Phosphorous solubilization activity of the isolated strains (**A**) GI-1; (**B**) GI-6.

The phosphorus solubilization test showed positive results for three isolates (GI-1, GI-6, and S-5) out of five. Endophytic strains expanded on the medium for over 24 h and resulted in hallow zones around each colony, providing evidence of the isolated strain’s ability to use the inorganic phosphate present in the medium. This test was completed over 8 to 10 days. Zone formations could be seen with the naked eye. Isolated Siran was used as a negative control. All the three positive isolates developed a clear zone with a diameter between 1.5 and 3.5 mm. The solubilization index was calculated by the formula: SI = clearing zone + colony diameter/colony diameter. Isolate Ghaneemat IBGE formed the maximum solubilization zone (3.5 mm) (Figure 8, Table 2 and Supplementary Table S1).

#### 3.1.5. Nitrogen Assimilation Potential of the Isolates

Bacterial isolates AH-1, GI-6, and S-5 were positive for nitrogen assimilation, which caused a change in the medium. The transformation of a clear medium to a turbid one was regarded as a success. Isolate AH-1 had the best result, followed by isolate GI-6. Positive isolates were compared to a control which consisted of a nitrogen-free medium with no inoculum. The turbidity of a medium increased upon the expansion of tested isolates, thus implying higher nitrogen assimilation (Supplementary Figures S3 and S4, Table S2). A 23-day long period was necessary to obtain clear results.

#### 3.1.6. Antimicrobial Activity of the Isolated Strains against Pathogenic Bacteria

**Figure 9:**
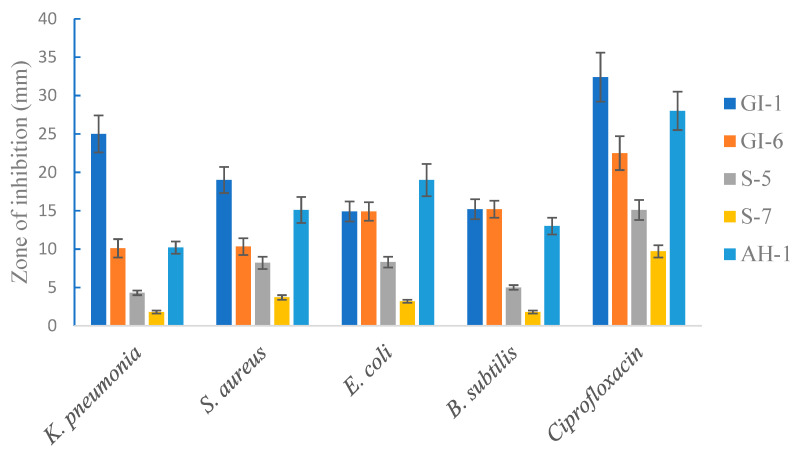
Antimicrobial activity of the isolated strains against selected pathogenic and non-pathogenic bacteria. Ciprofloxacin was used as a positive control. Data are averages ± SD values.

The selected endophytic bacteria from the three varieties of wheat seeds were studied to determine their in vitro inhibitory activity against the pathogenic bacteria *Klebsiella pneumonia*, *Escherichia coli*, and *Staphylococcus aureus*, and the non-pathogenic bacterium *Bacillus subtilis*. The five isolates (GI-6, GI, S. S-7, S-5, and AH-1) appeared to have a broad spectrum of antimicrobial activity in vitro. Indeed, all the analyzed endophytic strains exhibited substantial growth inhibition of the pathogenic bacterial strains, while Ciprofloxacin showed the highest inhibitory effect. The highest antimicrobial activity among the tested strains was determined with the isolates of GI-6 against *K. pneumonia*, while the second strongest inhibitory effect of GI-6 was against *E. coli*. The isolate S-7 showed the least antimicrobial activity among the studied isolates against the pathogenic bacterium *B. subtilis*. The different doses of all the isolates extracted against the tested microbes were observed to produce varying zones of inhibition ranging from 25 mm to 5 mm (Figure 9).

### 3.2. Screening of Endophytic Isolates for Drought and Salinity Stress Resistance

**Figure 10:**
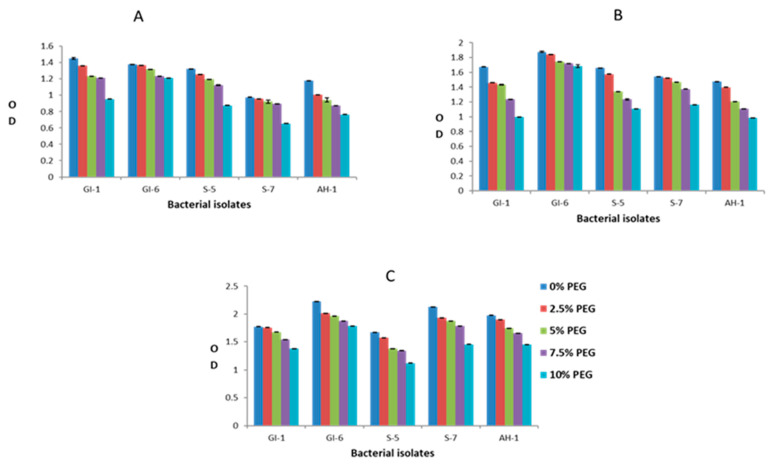
Bacterial growth, estimated by measuring the optical density (OD), against drought stress, at different poly ethylene glycol (PEG) concentrations, in the first three days: (**A**) day 1, (**B**) day 2, (**C**) day 3.

**Figure 11:**
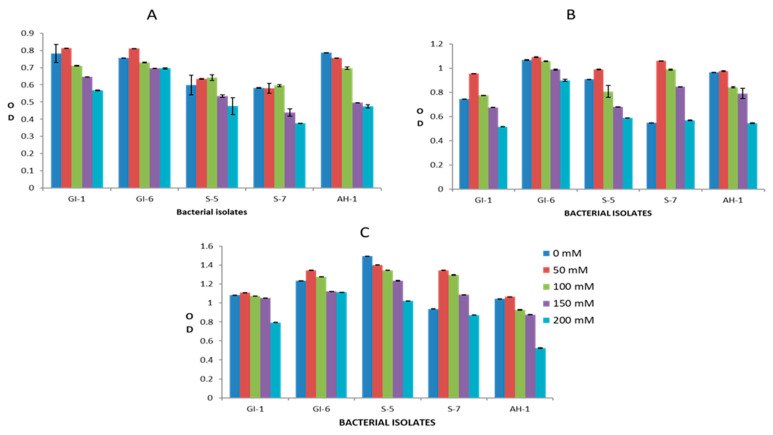
Bacterial growth, estimated by measuring the optical density (OD), against salinity stress, at different NaCl concentrations (0–200 mM), in the first three days: (**A**) day 1, (**B**) day 2, (**C**) day 3.

Overall, the analyzed bacterial isolates showed significant resistance to the adverse effects of both drought and salinity stress. The increasing PEG concentrations had a clear negative impact on the growth of bacterial strains GI-1, S-5, S-7, and AH-1, whereas the GI-6 strain was less affected by elevated PEG concentration. This bacterial strain growth remained continuous (OD value ranged from 1.2–1.4 on day 1, 1.6–1.8 on day 2, 1.7–2.2 on day 3) upon all applied PEG concentrations, suggesting resistance to drought stress, as indicated in Figure 10. Similarly, bacterial strain GI-6 maintained consistent growth upon all applied concentrations of salts in LB media (Figure 11). On the contrary, increasing salt concentration reduced GI-1, S-5, S-7, and AH-1 strain growth. Overall, the results revealed that the strain GI-6 possessed a much stronger capability to tolerate both drought and salt stress than the other tested strains.

### 3.3. GI-6 Isolate Growth-Promoting Effect on Wheat Plants 

**Figure 12:**
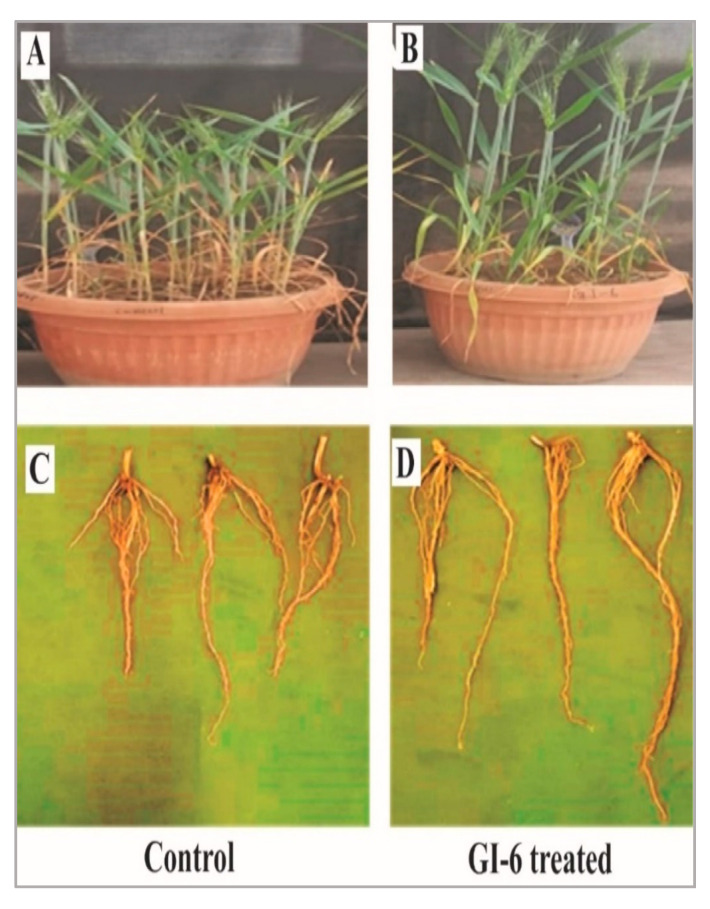
Phenotypic expression of the wheat variety Imdad plants: the height of plants inoculated with the endophytic strain GI-6 was increased (**B**) compared to non-inoculated control plants (**A**). Root length of GI-6 treated plants (**D**) was also significantly higher than non-inoculated control plants (**C**).

Seeds inoculated with GI-6 endophyte showed a dramatic increase in overall plant development compared to control plants. Most of the studied growth parameters were significantly (*p* ≤ 0.05) higher in the inoculated plants than in the non-inoculated controls (Table 3). Plant height was increased in the inoculated Imdad wheat variety plants (35.5 ± 1.08 cm; *p* = 0.02), compared to the control (27 ± 0.86 cm). The number of grains per spike and root length were significantly higher for the inoculated plants (15 ± 1.08; *p* < 0.05 and 13.3 ± 1.69 cm; *p* < 0.05, respectively) than the control (Table 3). Other growth parameters also increased in the inoculated plants, though the differences with the control were minor. The different growth parameters of inoculated and non-inoculated plants are illustrated in Figure 12.

## 4. Discussion

In the present study, endophytic bacteria were isolated from the seeds of different wheat varieties with the aim of evaluating their potential application in the development of bio-fertilizers. Five different bacterial strains designated as AH-1, S-5, S-7, GI-1, and GI-6 were isolated from three wheat varieties, i.e., Atta-Habib, Siren, and Ghaneemat-IBGE. Phylogenetic analysis showed that the sequences from the isolated endophytic bacterial strains had close homologies with *Bacillus altitudinis*, *B. aryabhattai*, *B. wiedmannii*, *Pseudomonas aeruginosa*, and *Burkholderia gladioli*. Several previous studies reported on the isolation of these bacterial species from several plants [29] such as rice, cotton, cucumber, sugar beet and potatoes. These endophytes have previously shown plant growth-promoting traits, such as IAA production, combined with siderophores, catalase, phosphate solubilization, and antimicrobial effects [30]. Similarly, the bacterial strains isolated in the present study showed several clear plant growth-promoting traits.

The catalase activities of endophytic bacterial strains obtained from wheat seeds in this study were different. Wheat endophytic bacteria had previously been found to be catalase positive [31]. In addition, a previous study found catalase production in 87.59% of bacterial strains isolated from a medicinal plant, *Ocimum* sp., endophyte [32].

All the analyzed endophytic strains showed IAA production, with the two strains GI-1 and GI-6 designated as *Pseudomonas aeruginosa* and *Burkholderia gladioli*, isolated from Ghaneemat-IBGE exhibiting maximum IAA production. Several studies have shown the ability of *P. aeruginosa* and *B. gladioli* to produce high concentrations of IAA, which have led to these two bacterial species being considered important endophytes for directly promoting the growth of associated plants [33]. Indeed, IAA is the most important phytohormone that directly promotes the growth of plants and microbes. Root growth and root length can be increased by endophytic bacteria with IAA-producing ability, resulting in a greater root surface area, which allows the plant to acquire more nutrients from the soil [34]. Our results support previous findings that IAA generation by endophytic bacterial species is beneficial to plant growth.

From our analysis, all the bacterial strains developed siderophores. However, qualitative and quantitative methods revealed that the three isolates, GI-1, GI-6, and S-5, had comparatively higher siderophore concentrations. Under various iron conditions, quantitative examinations of the siderophores were performed. When there was no Fe (III) citrate in the medium, siderophore development was at its peak. Similarly, increased iron concentrations in the medium had a negative effect on the synthesis of siderophores in a previous study by Nair et al. [35]. Our findings agree with previous research that found an inverse relationship between different iron concentrations and siderophore production [36,37]. Iron is abundant in the soil, mainly in the form of insoluble Fe^3+^ oxyhydroxides that plants and microbes cannot utilize. Plant-associated microbes use ferric reductases to convert Fe^3+^ to Fe^2+^ or to solubilize it with extracellular Fe^3+^ chelators called siderophores, which are released when soil iron levels are low [38]. This soluble Fe^3+^−siderophore complex is accessible to both plants and microbes [39]. Siderophore formation has been previously observed in isolated PGP rhizobacteria such as *Bacillus* sp., *Pseudomonas* sp., and *Paenibacillus* sp. [40]. Under various iron conditions, the isolated strain *Paenibacillus polymyxa* SQR-21 developed siderophores [41].

In the current investigation, the bacterial isolates AH-1, GI-6, and S-5 were found positive for nitrogen assimilation, with AH-1 demonstrating the best result, followed by GI-6, which implies their ability to fix atmospheric nitrogen. One of the most abundant, necessary, and vital elements for plant growth and development is nitrogen. The ability to fix atmospheric nitrogen has been discovered in most plant growth-promoting bacteria, including *Bacillus*, *Pseudomonas*, and *Burkholderia* species. In some instances, the ability of endophytic bacteria to fix nitrogen specifically led to positive effects on inoculated target plants [42]. The latter study [42] reported an increase in the growth of lodge pole pine inoculated with *P. polymyxa* (P2b-2R) in N-deficient soil. Furthermore, it has been demonstrated that the nitrogen fixation and growth promotion of canola (*Brassica napus* L.) were achieved by inoculation with the strain P2b-2R of *P. polymyxa* [43].

Phosphate solubilization capacity was found in our isolated strains. This capacity was particularly strong in the two strains GI-6 and S-5. Solubilization of phosphate is one of the main characteristics of endophytic strains that enhance the growth of associated plants, according to previous research [44]. In the latter study, the ability to dissolve phosphate was identified in *Paenibacillus* spp. strains from both mycorrhizal and non-mycorrhizal cucumber plants.

Endophytic bacteria possess the ability to inhibit the growth of pathogenic bacteria and fungi. Several endophytic bacteria have previously shown antagonistic effects against plant pathogens [45]. Similar results were reported in our study, where 4 out of 5 analyzed bacterial endophytes (GI-1, GI-6, AH-1, and S-5) showed clear antimicrobial activity against tested pathogenic bacteria in a dose dependent manner. The highest antibacterial activity was observed for GI-6 against *Klebsiella pneumonia*. A previous investigation has reported a potent activity of Gladiolin, an antibiotic produced by *Burkholderia gladioli*, against *Mycobacterium tuberculosis* [46]. 

Salinity tolerance of the isolated endophytic strains was evaluated under elevated salt concentrations and detected in all five strains tested. In previous studies on endophytic microbes from desert-dwelling plants, high salt tolerance of bacterial endophytes was also observed [47]. Zhao et al. [48] have proposed that bacterial endophytes live and multiply within the plant where the salt concentration is relatively low due to osmotic stress and toxic ions. In this study, the endophytic bacterial strains showed utmost resistance to various concentrations of salt, which may be due to a neutralization effect. 

Due to their unpredictable and devastating impacts on plant function as a whole, abiotic stresses such as salt and drought have been highlighted as key variables that impede food production [49,50]. Under limited irrigation, endophytic bacterial strains *Bacillus pumilus* and *B. subtilis* can help plants to develop faster [49]. Moreover, the use of bacterial endophytes in enhancing tomato growth under salt stress has been reported [51]. We tested the plant growth-promoting characteristics of our isolated strain GI-6 in the inoculation experiments, which involved seeds of the wheat variety Imdad. The inoculated plants showed better growth compared to that of the non-inoculated control plants. Some growth parameters were significantly improved in the inoculated plants than in the control plants. Similar results were produced when endophytic strains of *Bacillus velezensis* and *B. stratosphericus* were applied to plants of *Lilium* [21,42]. The latter studies reported that the improved growth of *Lilium* plants upon inoculation with the endophytes might be due to the plant growth-promoting potential of the inoculated bacteria. Similarly, in the present study, the growth improvement of inoculated wheat plants might be due to the biochemical and growth promoting effects of the endophytic strain GI-6.

## 5. Conclusions

We performed a comprehensive phylogenetic and biochemical analysis of five endophytic bacterial strains isolated from the seeds of 3 different wheat varieties. The analyzed strains, belonging to the genera *Bacillus*, *Pseudomonas* and *Burkholderia*, showed different levels of catalase activity, nitrogen assimilation, phosphate solubilization, and the ability to produce IAA and siderophores, which makes them strong candidates for exploitation as plant growth-promoting microbes. We found antimicrobial activity against pathogenic bacteria in all the studied strains, thus showing their potential effect to enhance disease-resistance in plant of high economic value. Moreover, the analyzed endophytic bacteria showed significant resistance to drought and salinity stress, which are well known issues in agrobusiness, causing a critical loss of crop yield every year [52]. Among all the investigated bacterial isolates, the most promising strain for application in sustainable agriculture was GI-6, which showed a clear growth-promoting effect on the inoculated wheat plants. The endophytic microbes analyzed in our study may represent an important, environmental-friendly alternative to chemical fertilizers and pesticides to enhance plant growth and yield under environmental stress, thus supporting the development of sustainable agriculture.

## Figures and Tables

**Table 1:** Identification of the endophytic bacteria isolated from wheat varieties.

Bacterial Strains	Wheat Variety	BLAST Matches	Top-Hit Strain with Accession Number	Similarity (%)	Completeness (%)
AH-1	Atta-Habib	*Bacillus altitudinis*	41KF2b(T) ASJC01000029	99.97	100
S-5	Siren	*Bacillus aryabhattai*	B8W22(T) EF114313	100.0	100
S-7	Siren	*Bacillus wiedmannii*	FSL W8-0169(T) LOBC01000053	100.0	100
GI-1	Ghaneemat-e-IBGE	*Pseudomonas aeruginosa*	JCM 5962(T) BAMA01000316	99.80	100
GI-6	Ghaneemat-e-IBGE	*Burkholderia gladioli*	NBRC 13700 (T) BBJG01000151	99.70	100

**Table 2:** Phosphorous solubilization activity of the isolated strains.

Isolates	Halo Zone Diameter (mm)	Colony Diameter (mm)	Solubilization Index
GI-1	2.90 ± 0.21	1.00 ± 0.05	2.90
GI-6	3.50 ± 0.26	1.10 ± 0.08	4.18
S-7	3.10 ± 0.18	1.85 ± 0.12	2.65
S-5	3.50 ± 0.24	1.10 ± 0.09	4.18
HA-1	2.70 ± 0.14	1.10 ± 0.10	3.45

**Table 3:** Plant growth promotion in wheat variety Imdad plants inoculated with GI-6 bacterial strain.

Treatment	Plant Height (cm)	Spike Length	Leaf Length	Root Length	Number of Grains per Spike
Control plants	27.0 ± 0.82 ^a^	5.8 ± 0.64 ^a^	10.4 ± 0.94 ^a^	8.06 ± 2.05 ^a^	12.0 ± 0.87 ^a^
Inoculated plants	35.5 ± 1.08 ^b^	7.0 ± 1.63 ^a^	15.0 ± 0.81 ^b^	13.3 ± 1.69 ^a^	15.0 ± 1.08 ^a^

Means are averages ± SD (*n* = 5). Values in a column with different letters ‘^a^’ and ‘^b^’ are significantly different by Student’s *t* test at (*p* ≤ 0.05). Controls are non-inoculated plants.

## Data Availability

Data presented in this study can be found as part of the manuscript, and in the Supplementary Materials.

## Supplementary Materials

The following are available online at https://www.mdpi.com/article/10.3390/microorganisms10010021/s1, Table S1: Biochemical characterization of the endophytic bacteria isolated from different varieties of wheat, Table S2: Nitrogen assimilation activity of isolated strains, Figure S1: Catalase activity of the isolated strains, Figure S2: IAA production activity of the isolated strains, Figure S3: Nitrogen assimilation of isolated strains on Ashby N free media, Figure S4: Nitrogen assimilation of isolated strains on Jensen N free agar media.
