# Supplementary material for: Molecular and Biochemical Characterization, Antimicrobial Activity, Stress Tolerance, and Plant Growth-Promoting Effect of Endophytic Bacteria Isolated from Wheat Varieties
Source: Microorganisms. 2021 Dec 23;10(1):21. doi: 10.3390/microorganisms10010021 (PMC8777632; doi:10.3390/microorganisms10010021)
Supplement: Supplementary file 1 [file microorganisms-10-00021-s001.zip › microorganisms-1499355-supplementary.pdf]

Molecular and Biochemical Characterization, Antimicrobial Activity, Stress Tolerance, and Plant Growth-Promoting Effect of Endophytic Bacteria Isolated from Wheat Varieties

Dawood Shah, M. Sayyar Khan Kazi, Shahkaar Aziz, Haidar Ali, and Lorenzo Pecoraro

Table S1. Biochemical Characterization of the endophytic bacteria isolated from different varieties of wheat.

Isolated Strains	Catalase Activity	IAA Production	Phosphate Solubilization	Siderophore Production
**GI-1**	+	+++	+	+
**GI-6**	+++	++	++	++
**S-5**	++	+	+	-
**S-7**	++	+	-	-
**AH-1**	-	-	-	-

− Absence of activity, + less activity, ++ moderate activity, +++ high activity

Table S2. Nitrogen assimilation activity of isolated strains.

Tested Medium	Isolates				
	GI-1	GI-6	S-5	S-7	AH-1
**Jensen N free agar media**	+	++	+	-	+++
**Ashby N free media**	-	++	+	-	+++

− Absence of activity, + less activity, ++ moderate activity, +++ high activity

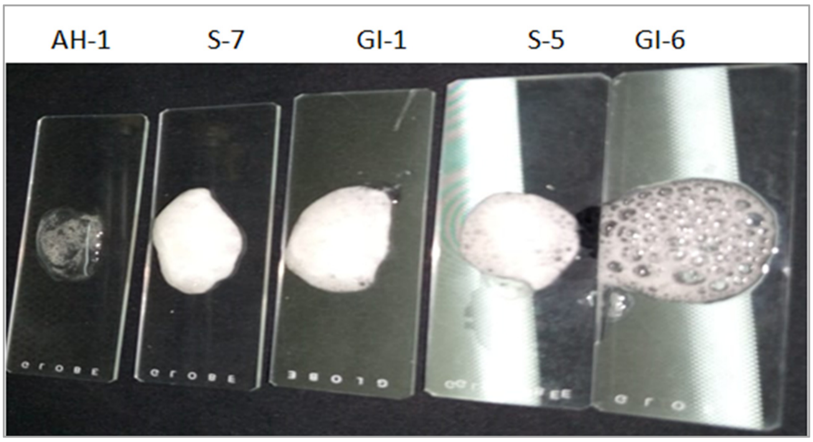

Figure S1. Catalase activity of the isolated strains.

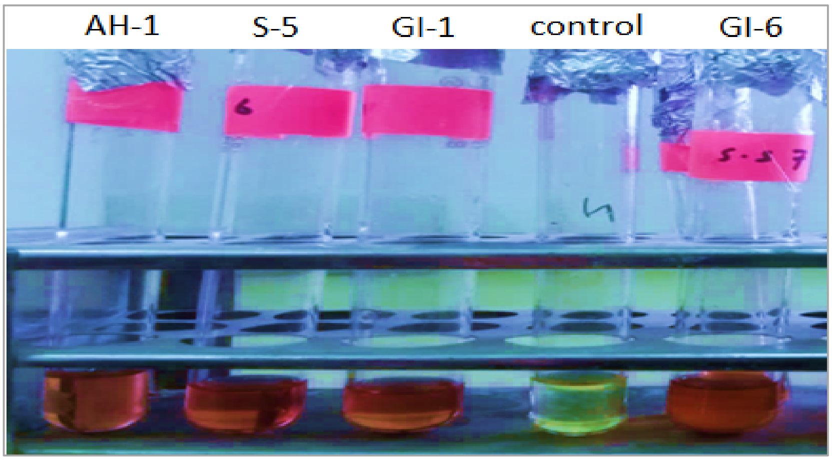

Figure S2. IAA production activity of the isolated strains

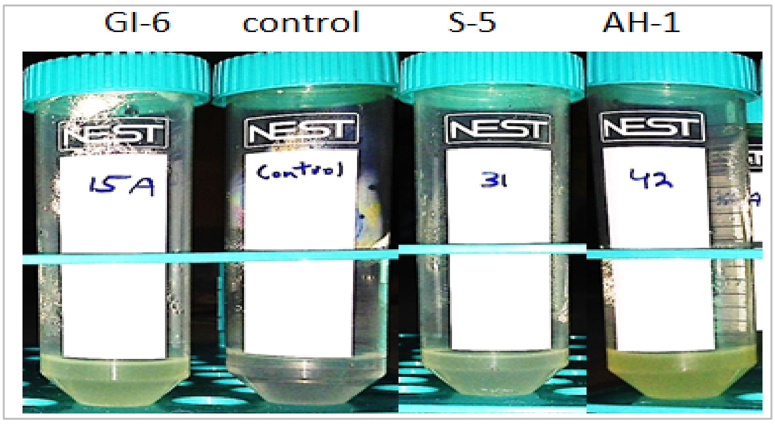

Figure S3. Nitrogen assimilation of isolated strains on Ashby N free media

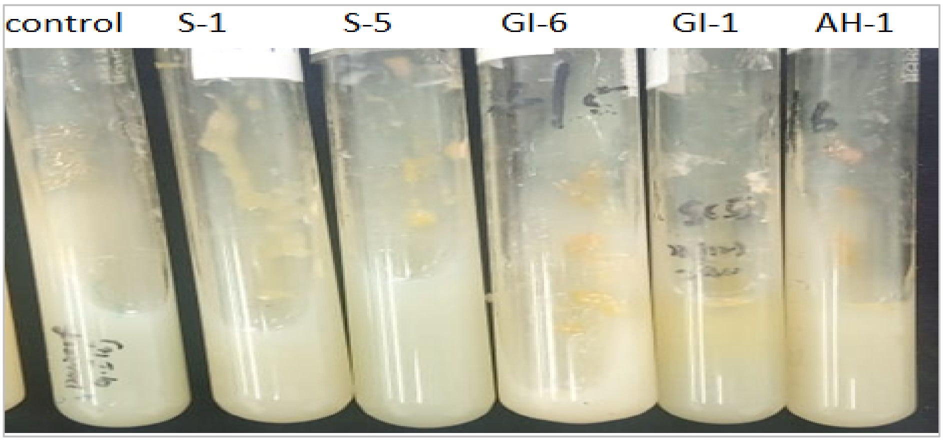

Figure S4. Nitrogen assimilation of isolated strains on Jensen N free agar media
